# Analyzing social media for measuring public attitudes toward controversies and their driving factors: a case study of migration

**DOI:** 10.1007/s13278-022-00915-7

**Published:** 2022-09-10

**Authors:** Yiyi Chen, Harald Sack, Mehwish Alam

**Affiliations:** 1 FIZ Karlsruhe - Leibniz Institute for Information Infrastructure, Karlsruhe, Germany; 2 Institute for Applied Informatics and Formal Description Systems (AIFB), Karlsruhe Institute of Technology, Karlsruhe, Germany

**Keywords:** Knowledge base, Social media analysis, Public attitudes, Hate speech detection, Immigration attitudes

## Abstract

Among other ways of expressing opinions on media such as blogs, and forums, social media (such as Twitter) has become one of the most widely used channels by populations for expressing their opinions. With an increasing interest in the topic of migration in Europe, it is important to process and analyze these opinions. To this end, this study aims at measuring the public attitudes toward migration in terms of sentiments and hate speech from a large number of tweets crawled on the decisive topic of migration. This study introduces a knowledge base (KB) of anonymized migration-related annotated tweets termed as MigrationsKB (MGKB). The tweets from 2013 to July 2021 in the European countries that are hosts of immigrants are collected, pre-processed, and filtered using advanced topic modeling techniques. BERT-based entity linking and sentiment analysis, complemented by attention-based hate speech detection, are performed to annotate the curated tweets. Moreover, external databases are used to identify the potential social and economic factors causing negative public attitudes toward migration. The analysis aligns with the hypothesis that the countries with more migrants have fewer negative and hateful tweets. To further promote research in the interdisciplinary fields of social sciences and computer science, the outcomes are integrated into MGKB, which significantly extends the existing ontology to consider the public attitudes toward migrations and economic indicators. This study further discusses the use-cases and exploitation of MGKB. Finally, MGKB is made publicly available, fully supporting the FAIR principles.

## Introduction

Measuring public attitudes toward a controversial issue such as war, COVID-19, migration, and climate change has become one of the mainstream challenges in social sciences. These attitudes can be measured with the help of surveys as well as interviews with specific individuals (Dennison and Drazanova 2018; Drazanova 2020). However, only a limited amount of data can be collected, processed, and analyzed in such a case. On the other hand, social media has become one of the most widely used and essential channels for the public to express their opinions about events around the globe.

Furthermore, migration has become one of the mainstream controversial topics in developed countries due to its effects on their culture, economy, demographics (such as age, gender and distribution). Many efforts have been put into studying the attitudes of the public toward migrations from various perspectives based on survey data (Hainmueller and Hopkins 2014; Dennison and Drazanova 2018; Helen Dempster and Hargrave 2020). This study, in particular, focuses on analyzing the social media platform Twitter to quantify and study public attitudes toward migrations and identify different factors that could be probable causes of these attitudes. Since the study mainly focuses on analyzing Twitter data, many kinds of challenges arise, i.e., millions of tweets in noisy natural language are being posted around the globe about a particular topic each day, which makes it impossible for humans to process this information, leading to the necessity of automated processing.

This paper focuses more explicitly on proposing a framework that measures public attitudes toward a chosen controversial issue, i.e., migration. Within the framework, MigrationsKB (MGKB) is constructed to achieve the following goals of the case study: (i) providing a better understanding of public attitudes toward migrations, (ii) explaining possible reasons why these attitudes toward migrations are what they are, (iii) defining a KB called MGKB built by taking into account the semantics underlying this field of study, (iv) defining possible scenarios where it can be applied, (v) and publishing this resource using FAIR principles (Wilkinson et al. 2016), i.e., make the resource Findable, Accessible, Interoperable, and Reusable (FAIR).

In order to study the public attitudes toward migrations as well as their drivers, the current study utilizes advanced artificial intelligence (AI) methods based on knowledge graphs and neural networks. The geotagged tweets are extracted using migration-related keywords to analyze public attitudes toward migrations in the destination countries in Europe. The irrelevant tweets are then filtered by using the state-of-the-art neural network-based topic modeling technique Embedded Topic Model (Dieng et al. 2020). It further utilizes contextualized word embeddings (Liu et al. 2020) and transfer learning for sentiment analysis and attention-based convolutional neural networks and bidirectional long-term short memory for hate speech detection. Temporal and geographical dimensions are then explored to measure public attitudes toward migrations at a specific time in a specific country. Entity linking is applied to identify the entity mentions linked to Wikipedia and Wikidata to enable easy search over the tweets related to a particular topic. In order to identify the potential social and economic factors driving the migration flows, external databases, such as Eurostat (2021) and Statista(O’Neill 2021), are used to analyze the correlation between the public attitudes and the established social and economic indicators (i.e., unemployment rate, disposable income, etc.) in a specific country in a certain period. The analysis aligns with the hypothesis that the countries with more migrants have fewer negative and hateful tweets. Such kind of analysis can help provide an overview of the countries that are more welcome to migrants. Further analysis is provided in Sect. 5 .

In order to enable reusability of the analysis results, the outcome is then integrated into MGKB, which is an extension of the ontology as initially defined in TweetsKB (Fafalios et al. 2018). It is extended by defining new classes and entities to cover the geographical information of the tweets, the results of hate speech detection, and integrating the information about the social and economic indicators that could be the potential cause of negativity or hatred toward migrants. Using the populated MGKB and social and economic indicators, a detailed analysis of potential factors affecting the public attitudes toward migrations is conducted. Finally, the use cases and scenarios are defined, and the answers can be retrieved with the help of SPARQL queries. The source code has been made publicly available for reproducibility reasons via GitHub.^1^ Information related to MGKB is available through the web page.^2^ MGKB is query-able via a SPARQL endpoint,^3^ and the dump of annotated data is available at Zenodo.^4^

This paper is structured as follows: Sect. 2 discusses the related work. Sect. 3 details how the resource is generated, while Sect. 4 presents ontology underlying MGKB. Sect. 5 presents a detailed analysis of economic/social factors affecting the public attitudes toward migrations. Sect. 6 discusses some use cases, scenarios, and sustainability of MGKB. Finally, Sect. 7 concludes the paper and gives an insight into future work.

## Related work

This section discusses studies that combine KBs and Twitter information belonging to various domains. It then discusses studies that analyze migration-related social media data. Finally, an insight into the studies assessing the public attitudes toward migrations is presented.

### Knowledge bases based on Twitter data

Several studies have been conducted that provide a KB containing Tweets from a particular time for making it more usable by researchers. TweetsKB contains more than a 1.5 billion tweets spanning more than 7 years (Feburary 2013–December 2020), including entity and sentiment annotations. It provides a publicly available RDF dataset using established vocabularies to explore different data scenarios, such as entity-centric sentiment analysis and temporal entity analysis. In the event of the COVID-19 pandemic, TweetsCOV19 (Dimitrov et al. 2020) was released, which deploys the RDF schema of TweetsKB. It provides a KB of COVID-19-related tweets, building on a TweetsKB subset spanning from October 2019 to April 2020. The study applies the same feature extraction and data publishing methods as TweetsKB. Apollo (Alam et al. 2020b) is a visualization tool analyzing textual information in the geotagged Twitter streams of COVID-19-related hashtags using sliding windows, which performs sentiment and emotion detection of the masses regarding the trending topics of #COVID-19.

As a step forward in combining KB and Twitter information in the field of analyzing migration-related data, MigrAnalytics (Alam et al. 2020a) is introduced. It uses TweetsKB as a starting point to select data during the peak migration period from 2016 to 2017. MigrAnalytics analyzes tweets about migrations from TweetsKB and then further combines European migration statistics to correlate with the selected tweets. However, it uses a very naive algorithm for performing sentiment analysis, and it does not introduce any sophisticated way to remove irrelevant tweets. Most recently, dynamic embedded topic model (Dieng et al. 2019) is deployed to analyze tweets and capture the temporal evolution of migration-related topics on relevant tweets. The results are then used to extend the TweetsKB (Chen et al. 2021). In contrast, the methods used for generating MGKB are more advanced and recent in sentiment analysis, hate speech detection, and entity linking. The RDFS model is extended with relevant topics, as well as geographical information. Moreover, the social and economic factors extracted from external databases and the correlation analysis between the potential driving factors and semantic analysis output are conducted (cf. Sect. 5).

### Migration-related social media data analysis

With the ever-growing prolific user input on social media platforms, there have been many efforts in analyzing the data from social media networks, such as Twitter and Facebook, regarding the topic of migrations. In Zagheni et al. (2014), the authors use geolocated data for about 500,000 users in OECD^5^ countries from Twitter to infer international and internal migration patterns during May 2011–April 2013, while using a difference-in-difference approach to reduce selection bias of the Twitter data with the OECD population when inferring trends in out-migration rates for single countries. Another work (Hübl et al. 2017) uses geotagged tweets that focus on identifying and visualizing refugee migration patterns from the Middle East and North Africa to Europe during the initial surge of refugees aiming for Europe in 2015. In another study (Drakopoulos et al. 2020) leveraging the geoinformation of the Tweets, the authors use machine learning techniques to study Twitter’s political conversation about the negotiation process for the formation of the government in Spain between 2015 and 2016 over different cities, and the factors conditioning the debate are analyzed, such as demographics, cultural factors and proximity to the centers of political power. Recently, Armstrong et al. (2021) discusses the challenges when identifying migration from geolocated Twitter data. Furthermore, it concludes that the data used for analyzing migration patterns are highly skewed by the subpopulation “transnationals” (i.e., citizens who seemingly live in two or more countries simultaneously and seamlessly move across borders) rather than conventional classified migrants. The skewness of the data limits its utility in studying migration populations. In comparison, MGKB deals with text data in EU destination countries of refugees regardless of the origin countries and measures the attitudes toward migrations in general.

Focused on migration-related party communication on social media, Heidenreich et al. (2020) analyzes migration discourses from the official accounts of political parties on Facebook across Spain, the UK, Germany, Austria, Sweden, and Poland. The study concludes that political actors from extreme left/right parties address migration more frequently and negatively than the political players in the middle of the political spectrum. Instead of political actors, MGKB focuses on general public sentiments toward migration.

To analyze Twitter data over time, Aletti et al. (2021) presents a model to reproduce the sentiment curve of the tweets related to specific topics and periods, including the Italian debate on migration from January to February 2019, and to provide a prediction of the sentiment of the future posts based on a reinforcement learning mechanism (Aletti et al. 2020). The reinforcement learning mechanism is based on the most recent observations and a random persistent fluctuation of the predictive mean. While in Drakopoulos et al. (2021), the authors focus on determining which tweets cause multiple sentiment polarity alternations to occur based on a window segmentation approach and an offline framework for discovering and tracking sentiment shifts of a Twitter conversation while it unfolds. However, the sentiment analysis conducted in Aletti et al. (2021) uses polyglot (Chen and Skiena 2014) python sentiment module, and Drakopoulos et al. (2021) uses SentiStrength,^6^ which are lexicon and rule-based methods. In comparison, the language models trained for MGKB provide state-of-the-art sentiment analysis and hate speech detection models. MGKB facilitates the sentiment evolution over time concerning refugees (cf. Sect. 3.3).

### Public attitudes toward migrations

While the popularity of the topic of migration has risen dramatically over the last decade in Europe, many efforts have been invested in analyzing the public attitudes toward migrations from various aspects. For instance, Hainmueller and Hopkins (2014) is based on the studies conducted during the last two decades explaining public attitudes on immigration policy in North America and Western Europe. The authors investigate the natives’ attitudes toward immigration from political economy and political psychology perspectives. In Dennison and Drazanova (2018), the authors explore the academic literature and the most up-to-date data across 17 countries on both sides of the Mediterranean. The study summarizes theoretical explanations for attitudes toward immigration, including media effects, economic competition, contact and group threat theories, early life socialization effects, and psychological effects. It also concludes that in Europe, attitudes toward immigration are notably stable rather than becoming more negative. While (Helen Dempster and Hargrave 2020) emphasizes the factors of individuals’ values and worldviews. It states that individual factors (i.e., personality, early life norm acquisition, tertiary education, familial lifestyle, and personal worldview) have a more stable and strong impact on the person’s attitudes toward immigration than the influence of politicians and media. More recently, Coninck et al. (2021) researches to relate the quality and quantity of (in)direct intergroup contact to attitudes toward refugees, based on the contact hypothesis proposed by (Allport.^1954^). The hypothesis postulates that intergroup contact reduces prejudice between members of traditionally opposed racial groups (Ata et al. 2009; Barlow et al. 2012), which is reflected in the analysis of driving factors of public attitudes toward migrations in this study (cf. Sects. 5.2 and 5.3). In Dennison and Drazanova (2018), Helen Dempster and Hargrave (2020), and Coninck et al. (2021), the survey data are used exclusively, while for Hainmueller and Hopkins (2014), a comprehensive assessment of approximately 100 studies, including both survey and field experiment data, is conducted.

On the contrary, many analyses regarding public attitudes toward migrations are performed based on automated approaches. In Freire-Vidal and Graells-Garrido (2019), Twitter data are leveraged to characterize local attitudes toward immigration, with a case study on Chile, where the immigrant population has drastically increased in recent years. Lapesa et al. (2020) and Blokker et al. (2021) introduce a debate corpus specific to the immigration discourse, sourced from *Die Tageszeitung* in 2015, a major national German quality newspaper, using a semi-automatic procedure, which integrates manual annotation and natural language processing (NLP) methods. In comparison, MGKB focuses on the data from average Twitter users to reflect a more realistic public opinion regarding migrations. Most recently, instead of using news outlets as a data source, Rowe et al. (2021) uses Twitter to track the public sentiment regarding immigration during the early stages of the COVID-19 pandemic in Germany, Italy, Spain, the UK, and the United States (US). In conclusion, it finds no evidence of a significant increase in anti-immigration sentiment. However, in this study, we observe a slight increase, 2% and 1%, in the percentages of hateful tweets and negative tweets toward migrations from 2019 to 2020 during the early stages of the pandemic in the European destination countries, as shown in Fig. 8(left). While the figure also shows no significant increase in overall anti-immigration sentiment over the last decade. However, evidence of persistent anti-immigration sentiment is presented (as shown in Fig. 5). In Pitropakis et al. (2020), the authors collected and annotated an immigration-related dataset of publicly available Tweets in the UK, the US, and Canadian English and explored anti-immigration speech detection using language features. In our study, we have studied Twitter data across 11 EU countries spanning from 2013 to 2020, with the help of the topics and linked entities in the MGKB, the public sentiments toward migrations, including specific events, e.g., COVID-19, Syrian Civil War, over the last decade can be queried (cf. Sect. 6).

## Pipeline for constructing MigrationsKB

**Fig. 1:**
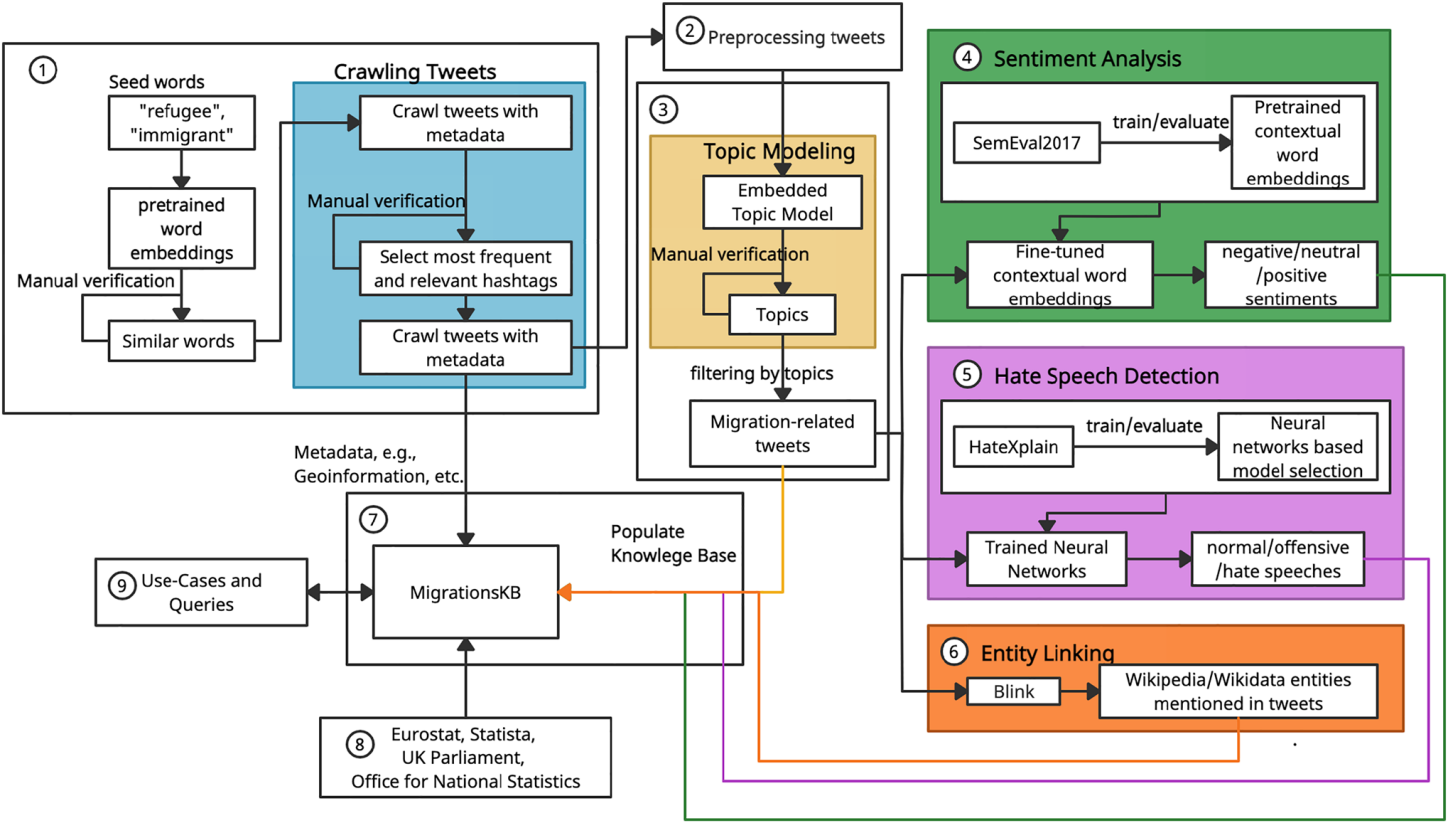
Pipeline for constructing MGKB

MGKB is an extension over TweetsKB with a specific focus on the topic of migration (as the name depicts). The goal of MGKB is: (i) to provide a semantically annotated, queryable resource about public attitudes on social media toward migrations, (ii) to provide an insight into which factors in terms of economic/social indicators are the cause of that attitude. In order to achieve these goals, a pipeline for constructing MigrationsKB is shown in Fig. 1. Step $\textcircled {1}$ is defining migration-related keywords and performing keyword-based extraction of geotagged tweets and their metadata. In step $\textcircled {2}$, the extracted tweets are preprocessed before further analysis. In step $\textcircled {3}$, topic modeling is performed for refining the tweets by removing irrelevant tweets crawled in the tweet extraction phase. Contextual Embeddings are then used for performing sentiment analysis in step $\textcircled {4}$. In order to further analyze the negative sentiments in terms of hate speech against the immigrants/refugees, tweets are further classified into three classes, i.e., hate, offensive, and normal, which is step $\textcircled {5}$. To enable search by mentioned entities in tweets, entity linking to Wikipedia and Wikidata is conducted in step $\textcircled {6}$. Furthermore, an analysis of factors causing the negative sentiment or the hatred against immigrants/refugees is performed with the help of visualization and statistical methods. In order to make this information queryable with the help of SPARQL queries, MGKB is constructed and populated with information extracted using the previously described steps (step $\textcircled {7}$). The statistics about these relevant factors, such as the unemployment rate and the gross domestic product growth rate (GDPR), are extracted from Eurostat, Statista, UK Parliament (Powell et al. 2021), and Office for National Statistics (Leaker 2021) (step $\textcircled {8}$). To facilitate applications in various fields, in step $\textcircled {9}$, the use-cases and queries are given in detail.

### Collecting migration-related tweets

In order to identify the public attitudes toward migrations in the EU countries, the first step is to select a list of destination countries, i.e., the countries hosting the immigrants/refugees. The statistics about asylum applications (annual aggregated) present on Eurostat is used to obtain the countries with a higher frequency of asylum applications during the period from 2013 to 2020. The list of countries includes Germany, Spain, Poland, France, Sweden, the United Kingdom (UK), Austria, Hungary, Switzerland, the Netherlands, and Italy.

In the second step, relevant tweets are extracted using keywords related to the topic of immigration and refugees using word embeddings. The words “immigration” and “refugee” are used as the seed words based on which top-50 most similar words are extracted using the pretrained Word2Vec model on Google News and fastText embeddings. These keywords are then manually filtered for relevance. Based on these keywords, the initial round of crawling the tweets is performed. Then, the crawled tweets are analyzed, and the most frequent hashtags, i.e., the hashtags occurring in more than 100 crawled tweets, are selected. These hashtags are verified manually for relevance and then used with the keywords for crawling tweets spanning from January 2013 to July 2021. The keywords and selected popular hashtags for filtering tweets are available on the GitHub repository.^7^ The 20 most frequently occurring hashtags containing “refugee” and “immigrant” are shown as the result of a query example on the web page.^8^

**Table 1:** Statistics of crawled and preprocessed tweets

Unique	Germany	Spain	Poland	France	Sweden	UK	Austria	Hungary	Switzerland	Netherlands	Italy	SUM
Crawled	26,892	21,392	6187	29,049	7556	265,448	6394	3355	12,062	16,095	30,023	424,453
Pre-processed	25,498	20,240	5764	26,514	7263	248,580	6027	3226	11,658	15,346	27,223	397,423

The extracted tweets are further filtered using their geographical information, i.e., only those tweets are selected which are geotagged with previously identified destination countries. About 66% of the crawled tweets have exact coordinates; the rest contain place names, such as “Budapest, Hungary.” The tweets are then pre-processed by expanding contractions, removing the user mentions, reserved words (i.e., RT), emojis, smileys, numeric tokens, URLs, HTML tags, stop-words, and punctuation marks. Moreover, the tokens except the hashtags are lemmatized. Eventually, the “#” in hashtags is removed, while the tokens in hashtags are reserved. Finally, the tweets of sentence length greater than one are retained. More specifically for topic modeling (cf. Sect. 3.2), words with document frequency above 70% are removed. Table 1 shows the statistics of the extracted and pre-processed tweets.

### Topic modeling

Since the tweets are collected based on keywords, many of them are irrelevant to the topic of migration. For example, some tweets are about the topic “migrant birds” or “Japanese Band Exile.” Due to the large number of tweets collected (i.e., 397,423 preprocessed tweets), it is hard to filter out irrelevant tweets manually. In order to automate this process, topic modeling is performed.

Topic modeling is used for extracting hidden semantic structures in textual documents. One of the classical algorithms for topic modeling is Latent Dirichlet Allocation (LDA) (Blei et al. 2003) which represents each topic as the distribution over terms and each document as a mixture of topics. It is a very powerful algorithm, but it fails in the case of a huge vocabulary. Therefore, for the current study, the most recent topic modeling algorithm, Embedded Topic Model (ETM) (Dieng et al. 2020), is chosen. Similar to LDA, ETM models each document as a mixture of topics, and the words are generated such that they belong to the topics (ranked according to their probability). It also relies on topic proportion and topic assignment. Topic proportion is the proportion of words in a document that belongs to a topic, which are the main topics in the document. Topic assignment refers to essential words in a given topic. In addition to that, ETM uses the embedding of each term and represents each topic in that embedding space. In word embeddings, the context of the word is determined by its surrounding words in a vector space, but in the case of ETM, the context is defined based on the topic. The topic’s distribution over terms is proportional to the inner product of the topic’s embedding and each term’s embedding.

In the current study setting, the word and the topic embeddings are trained on tweets. First, the word embeddings are generated by training a Word2Vec skip-gram model on all the preprocessed tweets for 20 epochs, with minimal word frequency 2, dimension 300, negative samples 10, and window size 4. For obtaining the optimal training parameters for ETM, its performance is computed on a document completion task (Rosen-Zvi et al. 2004; Wallach et al. 2009). The parameters for which the highest performance is achieved are selected, and the corresponding ETM model is utilized. In order to obtain optimal parameters, the dataset is split into 85%, 10%, and 5% for train, test, and validation sets, respectively. The size of the vocabulary of the dataset is 22850. The ETM is experimented with 25, 50, 75, and 100 topics to explore the optimal number of topics. Initialized with the pretrained word embeddings, the ETM is trained on training data, with batch size 1000, Adam optimizer, and ReLU activation function. In order to select the best number of epochs for training ETM, the model is trained repeatedly by selecting 1–200 epochs and evaluated on the task of document completion (as described previously). The model performs the best on 172 epochs with 50 topics.

**Table 2:** Results of ETM with different numbers of topics

# Topics	25	50	75	100
Val PPL	3329	3015	2920	**2870**
Best epoch	185	172	176	178
Topic coherence	0.0744	**0.0777**	0.0506	0.02
Topic diversity	**0.9696**	0.9288	0.9056	0.7832
Topic quality	**0.0721**	**0.0721**	0.0460	0.0157
Classified Nr. of topics	25	50	75	87

The bold numbers represent the best results for the metrics

The metrics topic coherence and topic diversity are used for evaluation (Dieng et al. 2020). Topic coherence provides a quantitative measure of the interpretability of a topic (Mimno et al. 2011), which is the average point-wise mutual information of two words drawn randomly from the same tweet. A coherent topic would display words that are more likely to occur in the same tweet. The most likely words in a coherent topic should have high mutual information. In contrast, *topic diversity* is defined as the percentage of unique words in the top 25 words of all topics. If there are topics that contain a high percentage of words that overlap with the words in another topic, i.e., the diversity would be low; then the topics are redundant. If the diversity is close to 1, the topics are diverse. The results for models with different numbers of topics are shown in Table 2. *Topic quality* is defined as the multiplication of topic coherence and diversity. The model with 100 topics has the lowest topic quality, and only 87 topics are assigned to the tweets, indicating redundancy of the topics. Comparing the models with 25, 50, and 75 topics, the model with 50 topics has the best topic quality and provides a wide range of topics. Therefore, the trained ETM model with 50 topics is used to classify the tweets’ topics.

**Fig. 2:**
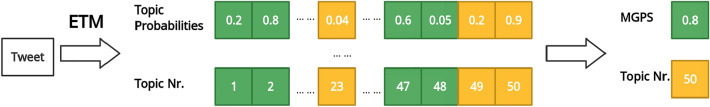
Filter tweets based on the MGPS score, and assign topic to tweet based on the maximal topic probability score. Green refers to migration-related topics, and orange refers to other topics

The tweets are then refined based on topic embeddings. For each topic, the top 20 words (ranked by their probability) are selected as representatives of the topic. These words are then manually verified based on their relevance to the topic of migration. Accordingly, migration-related topics are chosen. The migration-related tweets are selected with the help of the probabilities associated with all the topics. Regarding the chosen topics, the *maximal migration-related topic probability (MGPS)* score for each tweet is extracted, with which the threshold for reserving the tweets is set to 0.45 by manual evaluation. For example, in Fig. 2, the MGPS score of the tweet is 0.8, and therefore, the tweet is reserved. Moreover, the topic of each tweet is defined by the maximal probability of all the topics. As shown in Fig. 2, the topic of the tweet is 50, since it has the highest topic probability score. Consequently, out of the pre-processed 397,423 tweets, 201,555 are reserved for further analysis. In Table 3, topics 1, 2, and 3 belong to the chosen migration-related topics, while topics 4 and 5 are not. More specifically, the tweet “illegal immigrant get day uk free home cash health education maternity british national take fool kaitehopkins,” is classified as topic 4 and has the maximal probability score of 0.9598, which is over the threshold and is reserved for the MGKB. Topic words, migration-related topics,^9^ detailed plots, and statistics^10^ are publicly available on the web page.

**Table 3:** Example of topics, words belonging to the topics, an example Tweet, and its MGPS

Topic	Top words	Preprocessed tweet	MGPS
1*	Refugee, seeker, kill, alien, enter	Treatment refugee violate human rights dehumanize refugee endanger european value security argue group psychologist open letter	0.7195
2*	Great, call, immigration, question, town	Peddle lie interwoven thread brexit regional leave voter low exposure immigration easy scare foreigner queue town come assimilate quickly	1.1062
3*	Work, refugee, covid, border, woman	Yeah let corrupt nhs education system fine cause deport load hard work immigrant	0.8585
4	People, take, uk, health, hope	Illegal immigrant get day uk free home cash health education maternity british national take fool katiehopkins	0.9598
5	Stop, find, austria, future, country	Proven liar self promote cheat allow uncounted unchecked immigration country cause current crisis	0.4782

The topics with * are chosen as migration-related

**Fig. 3:**
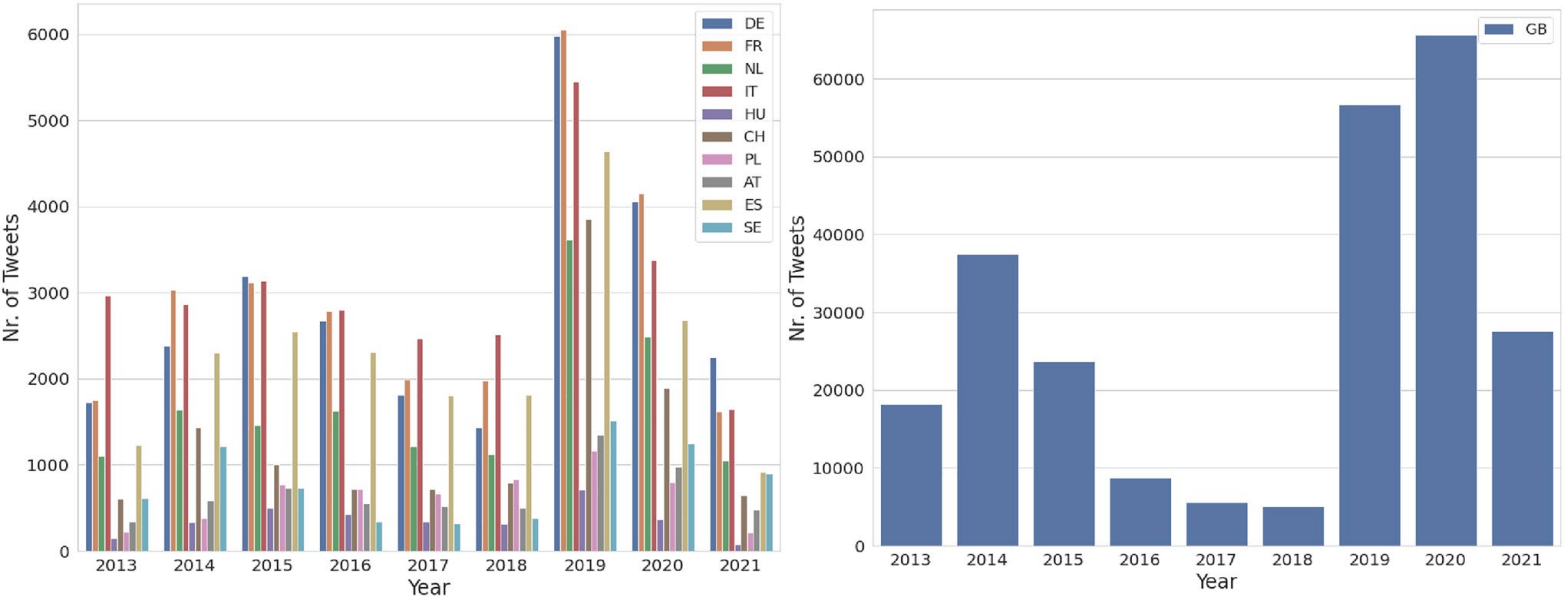
Distribution of all the Crawled Tweets based on geographic location

**Fig. 4:**
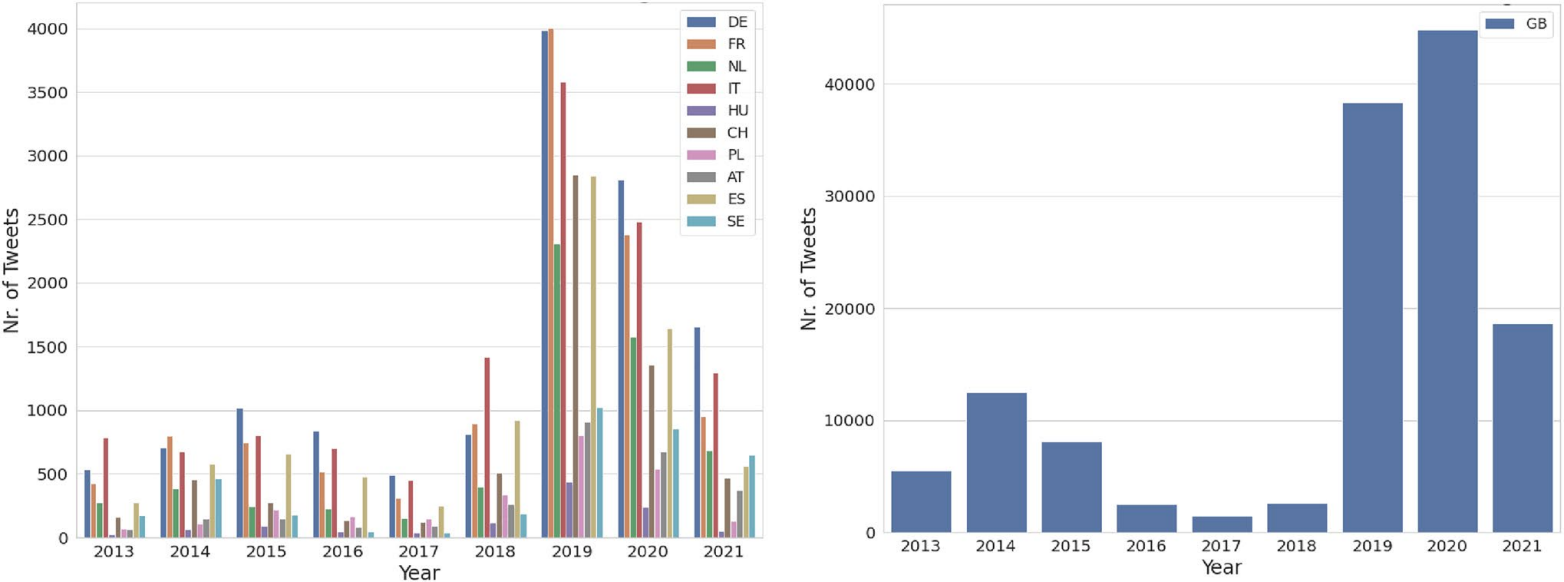
Distribution of Tweets based on geographic location after filtering using ETM

Fig. 3(left) shows the distribution of all the crawled tweets from 10 destination countries from 2013 to July 2021. Most of the tweets are from 2019 geotagged with Germany, France, the Netherlands, Italy, and Spain. Fig. 3(right) shows the distribution of all the crawled tweets with the geotag UK within the time frame from 2013 to July 2021. Most of the tweets are from the years 2019 and 2020. The UK is chosen because, currently, the focus of this study is the English language, and the majority of tweets are from there. Fig. 4 shows the distribution of the filtered tweets after using ETM. For all the countries, the graph shows similar proportions/trends as Fig. 3, but the number of tweets is lower than after filtering.

### Sentiment analysis

In order to measure the public attitudes toward migrations, sentiment analysis is performed by classifying the tweets into positive, negative, and neutral sentiments. These public sentiments in the destination countries are then analyzed based on their geographic location and temporal information.

Since there is a lack of datasets available for sentiment analysis, particularly in the domain of migration, the existing Twitter datasets for sentiment analysis are used for fine-tuning language models for transfer learning on the collected data. Two Twitter datasets for sentiment analysis are used most frequently, i.e., the Airline dataset^11^ and the SemEval2017 dataset (Rosenthal et al. 2017). The Airline dataset focuses on travelers’ opinions on Twitter, which is domain-specific. In comparison, the SemEval2017 dataset consists of broader topics of tweets, including a range of named entities (e.g., iPhone), geopolitical entities (e.g., Aleppo), and other entities (e.g., Syrian refugees, gun control, etc.). The language models are fine-tuned on both the datasets separately as well as on the combination. Table 4 shows statistics and results of Contextual Embedding Models on SemEval2017 test dataset. Bold values represent the best results.

**Table 4:** Statistics (a) and results (b) of contextual embedding models on SemEval2017 test dataset for sentiment analysis

	Train	Validation	Test	All	
(a)					
*SemEval2017*					
Negative	6291	752	766	7809	
Neutral	17,981	2256	2287	22,524	
Positive	15,833	2006	1960	19,799	
*Airline*					
Negative	7316	923	939	9178	
Neutral	2475	316	308	3099	
Positive	1921	225	217	2363	
*Combined*					
Negative	13,608	1736	1643	16,987	
Neutral	20,516	2535	2572	25,623	
Positive	17,693	2207	2262	22,162	

Model	Fine-tuned	Acc	$\hbox {F}_1$	Prec	Rec
(b)					
XLNet	SemEval2017	0.7066	0.6851	0.6988	0.6719
XLNet	Airline	0.5565	0.5987	0.5965	0.601
XLNet	–	0.3718	0.3482	0.3348	0.3627
BERT	SemEval2017	**0.7068**	**0.6949**	**0.7007**	**0.6892**
ULMFiT	SemEval2017	0.6624	0.6365	0.6342	0.6388
ULMFiT	Airline	0.4709	0.5215	0.52	0.5231
BERT	Airline	0.5117	0.5831	0.5736	0.5929
BERT	–	0.5417	0.5722	0.5753	0.5692
BERT	Combined	0.6691	0.6627	0.6484	0.6776

**Fig. 5:**
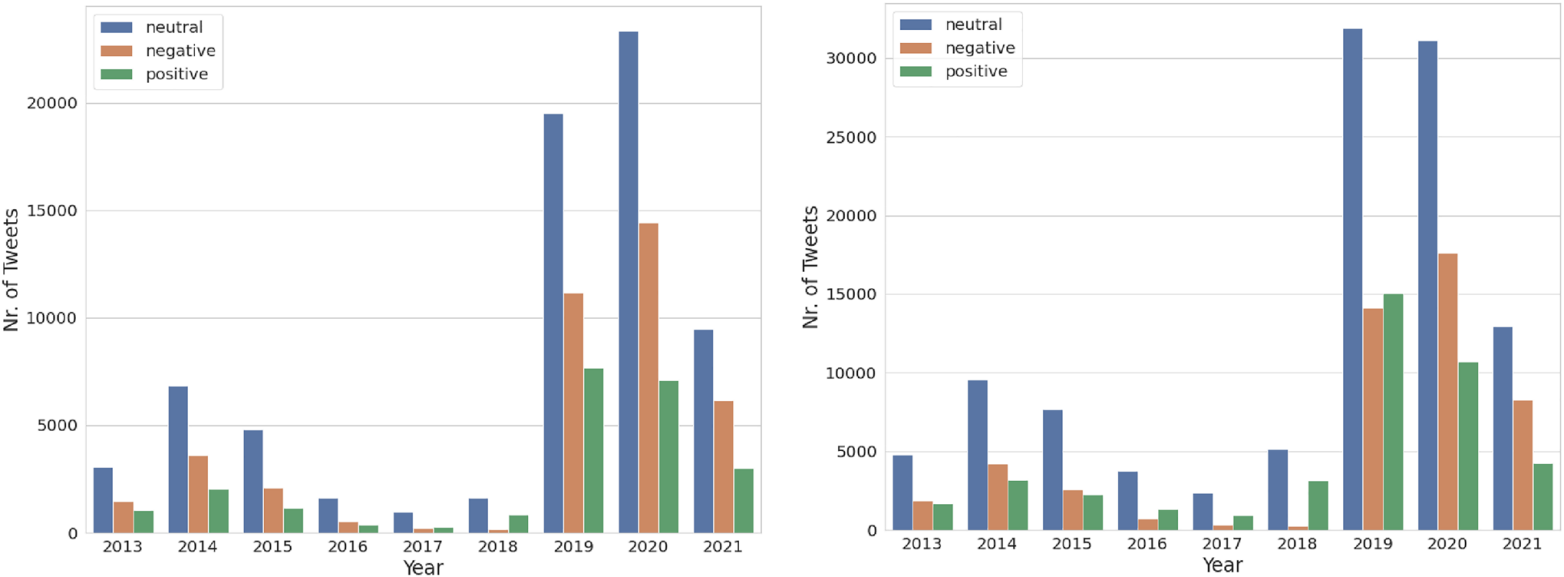
Temporal distribution of the sentiments of the public toward migrations. (Left) shows the sentiments of the people toward migrations in the UK, and (right) shows the sentiments for all 11 destination countries in Europe.

For transfer learning, three contextual embedding models are chosen, i.e., BERT (Devlin et al. 2019), XLNet (Yang et al. 2019), and ULMFit (Howard and Ruder 2018). These three models are fine-tuned, as mentioned earlier. The fine-tuned models are then tested on the test set of their corresponding datasets and the test set of the other datasets. The performance of each of these models is measured. Because the curated tweets obtained from previous steps are not domain-specific, the fine-tuned language model is required to perform well on a non-domain-specific dataset. Therefore, all the models are evaluated on the test set of SemEval2017 dataset. The results of all the models are shown in Table 4(b). As shown in Table 4(a), there are more neutral and positive tweets than negative ones in SemEval2017 which leads to class imbalance. The macro-metrics are more robust to class imbalance and reflect the real performance classifying the minority classes compared to micro-metrics. Hence, the macro—F$_1$ score, macro-precision, macro-recall, and standard accuracy are reported. Since BERT fine-tuned on SemEval2017 training dataset renders the best results, which is also the state-of-the-art on this dataset (Rosenthal et al. 2017), it is chosen for transfer learning on the collected tweets for sentiment analysis.

*Analyzing public sentiments toward migrations* In order to identify the public attitudes toward migrations, the sentiments of the tweets per country by year are aggregated. In Fig. 5a, the public sentiments in the UK from 2013 to July 2021 are shown. We observe that the total number of tweets regarding migration from 2013 to 2014 and the tweets with both positive and negative sentiments are increasing similarly. From 2016 to 2018, the topic of migration is less prevalent, while a sharp increase in the tweets regarding migration occurs from 2018 to 2020. Overall, the negative sentiment is more significant toward migrations than the positive sentiment. As shown in Fig. 5b, the public sentiment toward migrations in all 11 European destination countries follows similar trends as in the UK from 2013 to July 2021.

### Hate speech detection

To measure the negative attitude of the public toward migrations, hate speech detection is performed. The tweets are classified into one of three classes hate, offensive, and normal. We follow the definition from Davidson et al. (2017), which defines hate speech as the language that is used to express hatred toward a target group or is intended to be derogatory, humiliate and insult the members of the group. There are a lot of messages that are offensive but do not qualify as hate speech. One example from Vigna et al. (2017), the word “nigga” is used every day in online language by the African American community.

In order to perform transfer learning in this scenario, all the hate speech detection models are trained on recently published manually annotated data for hate speech detection, called HateXplain (Mathew et al. 2020). Similar to previous studies on hate speech detection, the sources of the dataset are Twitter (Waseem and Hovy 2016; Davidson et al. 2017; Founta et al. 2018) and Gab (Mathew et al. 2019). All the data are annotated using Amazon Mechanical Turk (MTurk) where each text is annotated based on: (1) whether it is hate speech, offensive speech, or normal; (2) the target communities in the text, including target groups such as Race, Religion, Gender, Sexual Orientation, and Miscellaneous; (3) if the text is considered hate speech or offensive speech by the majority, the annotators further annotate which parts of the text provide the rationale for the given annotation. (This ensures the explainability of manual annotation by the annotators.)

**Table 5:** The statistics (a) of the HateXplain Dataset and the results (b) of different hate speech detection models (Bold values show the best results)

Dataset	Normal	Offensive	Hateful	
(a)				
Train	6251	4384	4748	
Val	781	548	593	
Test	782	548	594	

Model	Acc	$\hbox {F}_1$	Prec	Rec
(b)				
BiGRU	0.6533	0.6353	0.6343	0.6364
BiGRU+Attn	0.6445	0.6344	0.6297	0.6392
BiLSTM	0.6284	0.6211	0.6169	0.6253
BiLSTM+Attn	0.6512	0.6421	0.6386	0.6457
CNN+GRU	0.6544	0.6545	0.6541	0.6549
CNN+GRU+Attn	0.6450	0.6330	0.6372	0.6436
CNN+BiGRU	0.6575	0.6489	0.6461	0.6517
CNN+BiGRU+Attn	0.6606	0.6472	0.6444	0.6501
CNN+BiLSTM	0.6372	0.6496	0.6523	0.647
**CNN+BiLSTM+Attn**	**0.6863**	**0.6751**	**0.6782**	**0.672**

**Fig. 6:**
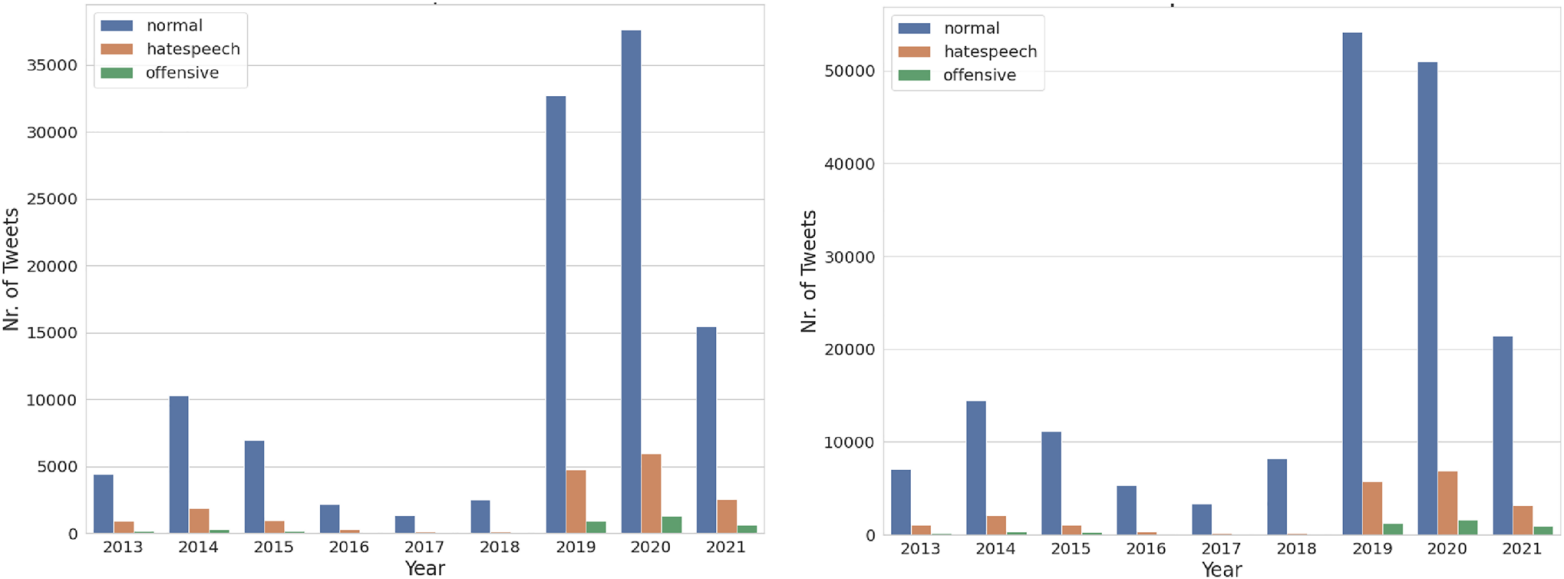
Temporal distribution of tweets after hate speech detection. (Left) shows the distribution of tweets from the UK, while (right) is for all the 11 EU countries

HateXplain is split into train, validation, and test dataset by 80, 10, and 10%, for which the stratified split is performed to maintain class balance. BiRNN (Schuster and Paliwal 1997) and BiRNN-Attention (Liu and Lane 2016) are widely used for text classification tasks, and CNN–GRU (Zhang et al. 2018) is used for hate speech detection. In the current study, the experimentation is conducted using combinations of various models from CNN, BiLSTM, GRU, BiGRU, and an attention layer for selecting the best model for hate speech detection. For all the models, pre-trained GloVe embeddings are used as reported in Mathew et al. (2020). A dropout layer with a dropout rate of 0.3 is applied after the word embedding layer. For CNN models, the convolutional layer has a filter size of 100 and window sizes 2, 3, and 4. The RNN models use hidden size 100. Finally, the softmax function is used for classifying the texts. The models with the highest accuracy on the validation dataset (after training on the training dataset) are chosen for test on the test dataset, whose results are reported in Table 5. Eventually, the best-performing pre-trained model CNN+BiLSTM+Attention, which is comparable to the results of the best performing model from Mathew et al. (2020), is used for transfer learning on the collected tweets.

*Analyzing hate speech in public opinions about migration* The results for hate speech detection are aggregated temporally and geographically to identify the public’s negative attitude toward migrations. As shown in Fig. 6, the number of hateful tweets is increasing from 2013 to 2014, decreasing from 2016 to 2018. It is then increasing again from 2019 to 2020 both in the UK and overall in 11 destination countries. In general, the proportion of offensive and hateful tweets is always less than the tweets belonging to the normal class. In summary, the percentage of tweets classified as hate speech in the UK from 2013 to 2020 amounts to 12.98%, while in 11 destination countries, it is about 9.36%.

### Entity linking

For entity linking BLINK (Wu et al. 2020) is used, which utilizes Wikipedia^12^ as the target KB. Based on fine-tuned BERT, BLINK uses a two-stage approach. In the first stage, BLINK retrieves the candidates in a dense space defined by a bi-encoder that independently embeds the context of entity mention and the entity descriptions. Then, in the second stage, each candidate is examined with a cross-encoder, concatenating the entity mention and entity text. BLINK outperforms state-of-the-art methods on several zero-shot benchmarks and also on established non-zero-shot evaluations such as TACKBP-2010 (Ellis et al. 2018). Out of 201555 tweets in MGKB, for 145747 tweets, there is at least one entity mention detected using BLINK. For one tweet, the maximum number of detected entity mentions is 30. In total, 89,076 unique entities are detected. Then, the entities from Wikipedia are mapped to the entities in Wikidata. The detected entities are available in the GitHub repository.^13^ Table 14 shows the query (a) and result (b) for retrieving a list of top 10 entity labels containing “refugee” and its frequency of detected entity mentions. As shown in the table, the entity “United Nations High Commissioner for Refugees” is the most frequent. With the help of mapped entities in Wikidata, and the semantic annotations from sentiment analysis and hate speech detection, more entity-centric analyses can be done, such as identifying the public attitudes toward certain groups, for example, refugees (cf. Sect. 6).

## MGKB Ontology

**Fig. 7:**
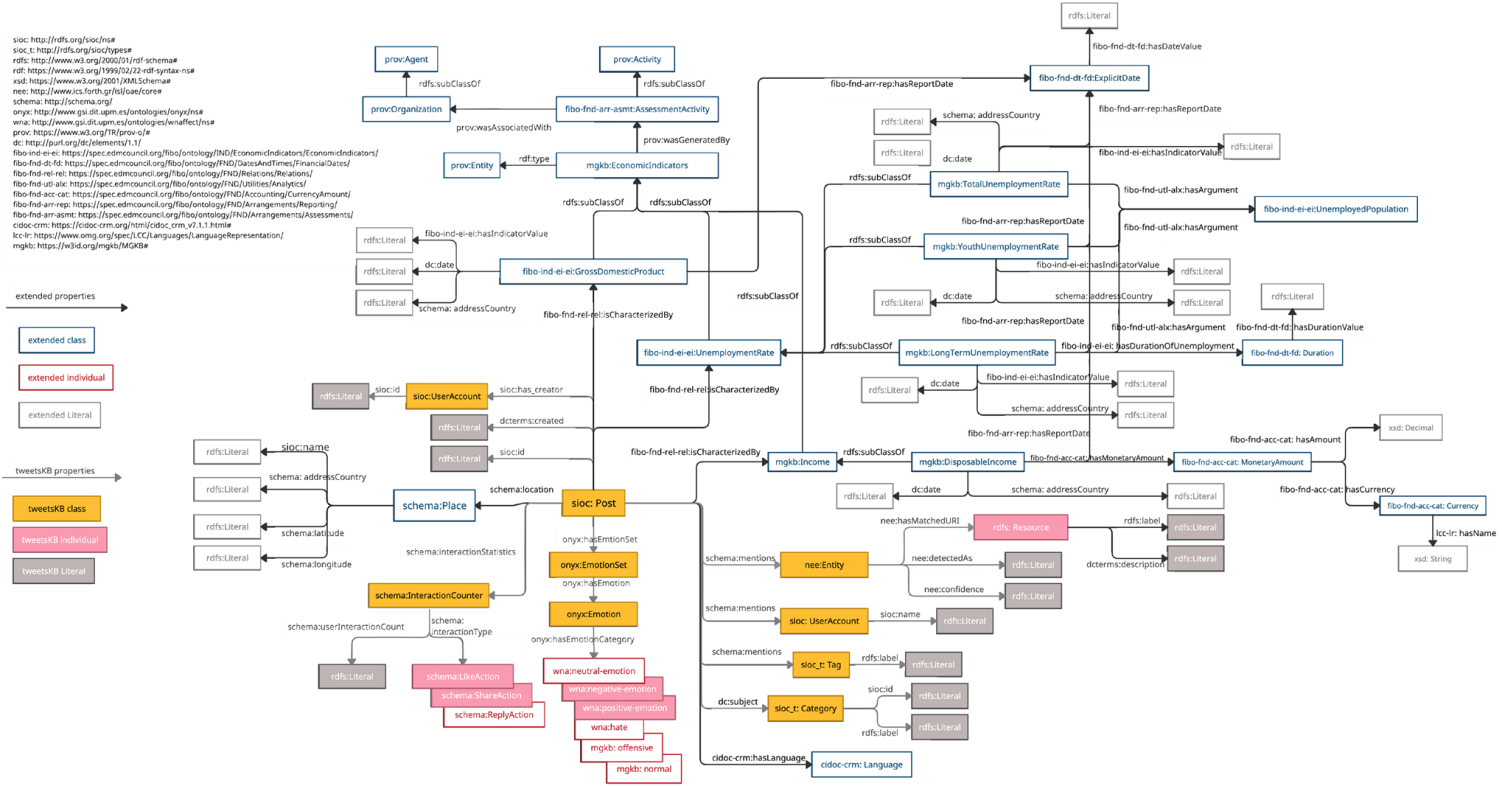
MGKB Ontology

This section discusses the extensions in the ontology of TweetsKB for incorporating public attitudes toward migrations, as well as the economic indicators which drive these attitudes. In order to fulfill the purpose of this study, several classes from already existing ontologies are re-used. Complete documentation of this ontology is available on the web page.^14^ A full ontology of MGKB is shown in Fig. 7.

For incorporating the metadata about the geographical location, following information is modified in the TweetsKB. The class schema:Place represents geographical information of a tweet. schema:location is used for associating a tweet (represented as sioc:Post) with schema:Place, i.e., its geographical information. sioc:name from SIOC Core Ontology (Berners-Lee et al. 1998; Bradner 1997) associates a place with its name represented as a text literal. schema:addressCountry specifies the country code of the geographic location of the tweet. schema:latitude and schema:longitude specify the coordinates of the geographical information. To enable search of topics of the tweets resulted from topic modeling (cf. Sect. 3.2), the class sioc_t: Category from SIOC Type Ontology (Berners-Lee et al. 1998; Bradner 1997) is used to represent the topics, whose property rdfs:label represents the top topic words and sioc:id refers to the id for the regarding topic.

### Representing economic indicators of EU

To represent the economic indicators of the destination countries obtained from Eurostat, Financial Industry Business Ontology (FIBO) (Bennett 2013) is used, and several new classes are defined in MGKB.

- The class fibo-ind-ei-ei:GrossDomesticProduct represents the GDPR of the country of the tweet in a certain year, which are specified by the properties schema:addressCountry and dc:date from DCMI, and the value of this indicator is represented by fibo-ind-ei-ei:hasIndicatorValue.
- The class fibo-ind-ei-ei:UnemploymentRate represents the unemployment rate in the country of the tweet in a certain year, represented with the help of the same properties, i.e., schema:addressCountry, dc:date, and fibo-ind-ei-ei:hasIndicatorValue.
- The class fibo-ind-ei-ei:UnemployedPopulation is used to specify the population of the unemployment rate.
- To represent the youth unemployment rate, total unemployment rate and long-term unemployment rate, the classes mgkb:YouthUnemploymentRate, mgkb:TotalUnemploymentRate and mgkb:LongTermUnemploymentRate are defined, which represent the unemployment rates with respect to the population, i.e., the youth unemployment population, the total unemployed population, and the population who are unemployed more than 12 months.
- To represent the amount of residential income, the class fibo-fnd-acc-cat:MonetaryAmount is used, which has a property fibo-fnd-acc-cat:hasCurrency, and the value of which is represented by the class fibo-fnd-acc-cat:Currency, having a property lcc-lr:hasName to indicate the name of the currency.
- mgkb:Income represents the income of residents in European countries.
- mgkb:DisposableIncome is a subclass of mgkb:Income, which represents the disposable net income per inhabitant in European with the help of property fib-fnd-acc-cat:MonetaryAmount.
- mgkb:EconomicIndicators represents the economic indicators, which has the subclasses fibo-ind-ei-ei:GrossDomesticProduct, fibo-ind-ei-ei:UnemploymentRate, and mgkb:Income.
- The class fibo-fnd-dt-fd:ExplicitDate represents the date when the statistics are last updated as a literal with the help of the property fibo-fnd-dt-fd:hasDateValue.
- The property fibo-fnd-rel-rel:isCharacterizedBy is used to associate a tweet with the social and economic indicators.

### Representing provenance information

To represent the provenance information about the economic indicators, i.e., Eurostat, Statista, UK parliament, and Office of National Statistics, PROV-O (Moreau and Missier 2013) is used. The class prov:Activity defines an activity that occurs over a period of time and acts upon entities, which are defined by the class prov:Entity. The class fibo-fnd-arr-asmt:Assessm-entActivity represents an assessment activity involving the evaluation and estimation of the economic indicators, which is a subclass of the class prov:Activity. The class prov:Organization represents a governmental organization or a company that is associated with the assessment activity, which is a subclass of the class prov:Agent. Further extensions are specified on the web page.

### Further extensions

Further extensions are made as follows to incorporate all the results and information from the pipeline:

- dc:subject represents a topic of a tweet resulting from topic modeling, which is represented by the class sioc_t:Category (cf. Sect. 3.2).
- wna:neutral-emotion represents the neutral sentiment of the tweet by applying sentiment analysis (cf. Sect. 3.3).
- wna:hate, mgkb:offensive and mgkb:normal represent the hate speeches, offensive speeches and normal speeches from hate speech detection of the tweets (see Sect. 3.4).
- schema:ReplyAction represents the action of reply regarding a tweet.

## Factors affecting the public attitudes toward migrations

In order to learn the potential cause of the negative public attitudes toward migrations, the factors such as unemployment rate, including the youth unemployment rate, long-term unemployment rate and total unemployment rate, real GDP growth rate, and disposable income, are studied and incorporated into MGKB (as discussed previously). These factors are identified by the experts (Dennison and Drazanova 2018) as the potential cause of negative attitudes toward migrations. These data are collected from Eurostat, Statista, UK Parliament, and Office for National Statistics.

### Correlation visualization

**Fig. 8:**
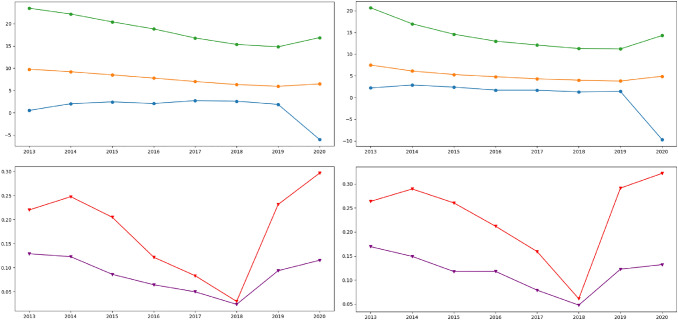
The trend of Negative sentiments and Hate Speech against immigrants/refugees in (left) all the identified destination countries and (right) the UK from 2013 to July 2021 (Green: youth unemployment rate (%), orange: total unemployment rate (%), blue: real GDP growth rate (%), red: negative tweets (%), purple: hate speech tweets (%)). (Color figure online)

Fig. 8 shows the comparison between the factors (such as youth employment rate, total employment rate, and real GDP growth rate) and the negative attitudes (i.e., negative sentiment and hate speeches) in all the extracted tweets. On average, in all 11 destination countries [see Fig. 8(left)] and individually in the UK [see Fig. 8(right)], the percentages of hate speech and negative sentiment of the public attitudes toward immigration are negatively correlated with the real GDP growth rate, and positively correlated with total/youth unemployment rate, from 2013 to 2018 and from 2019 to 2020. In 2019, the percentages of hateful and negative tweets are rapidly increased by more than 2% and 1%, respectively, compared to 2018. The analysis for each of the 11 countries is available on the website.

### Multivariate analysis of potential determinants

This section provides a more detailed and quantified analysis regarding various social and economic factors and their correlations with the public attitudes toward migrations, as facilitated by MGKB. One of the most influential frameworks in the study of intergroup relations and intergroup prejudice is the contact hypothesis (Broad et al. 2014), which indicates that greater intergroup contact can, under certain conditions, mitigate the prejudices, e.g., the prejudices of the local residents toward the immigrants.

**Table 6:** Correlation matrix of factors used in multivariate analysis of potential determinants (***$p<0.01$, **$p<0.05$, *$p<0.1$)

	Share negative	Share positive	Share offensive	Share hateful	Total unemployment	Long-term unemployment	Youth unemployment	Disposable income	Real GDP growth rate	Immigration flow per 100k population	First-time asylum applications per 100k population	Immigrant stock per 100k population
Share positive	− 0.75***	1										
Share offensive	0.75***	− 0.67***	1									
Share hateful	0.77***	− 0.52***	0.68***	1								
Total unemployment	− 0.1	0.19*	− 0.03	− 0.05	1							
Long-term unemployment	− 0.17	0.23**	− 0.05	− 0.06	0.95***	1						
Youth unemployment	− 0.02	0.11	0.05	0.04	0.94***	0.92***	1					
Disposable income	0.3**	− 0.11	0.1	0.26**	− 0.27**	− 0.32**	− 0.28**	1				
Real GDP growth rate	− 0.42***	0.14	− 0.27***	− 0.2*	− 0.11	− 0.06	− 0.14	− 0.4***	1			
Immigration flow per 100k population	− 0.08	0.06	− 0.25**	− 0.24**	− 0.33***	− 0.43***	− 0.48***	0.5***	− 0.01	1		
First-time asylum applications per 100k population	− 0.08	− 0.02	− 0.07	− 0.16	− 0.15	− 0.2*	− 0.16	0.01	0.13	0.31***	1	
Immigrant stock per 100k population	− 0.19	0.26**	− 0.32***	− 0.34***	− 0.06	− 0.07	− 0.18	0.7***	− 0.44***	0.71***	0.07	1

Table 6 shows an overview of the pairwise correlation in the panel dataset^15^ created from MGKB and external databases for other social and economic indicators, such as migrant stocks and the number of asylum applicants. Stars indicate correlation at the 1/5/10% significance level. By definition, the share of positive and negative sentiments is negatively correlated. Negative sentiment is positively correlated with the share of tweets that are classified as offensive or hateful. In the contrary, positive sentiment is negatively correlated with offensive and hateful speeches. Moreover, the share of offensive tweets is positively correlated with the share of hateful tweets. All the correlations mentioned above are significant at the 1% significance level. Regarding offensive and hateful content, in line with the contact hypothesis, there are negative correlations with both a higher immigrant stock and immigration flow. Moreover, real GDP growth rate is negatively correlated with both negative sentiments and offensive and hateful speeches. Overall, it shows that places with more migrants receive more migration and more first-time asylum seekers (all per 100k population, so that is not merely the effect of size). Also, in the countries where more migrants reside, there is more disposable income per inhabitant.

Moreover, Table 6 shows that the total unemployment rate, long-term unemployment rate and youth unemployment rate are highly positively correlated, among which total unemployment rate is the most representative indicator of the unemployment rates. Therefore, the total unemployment rate will be used for further analysis. Although disposable income has a correlation with the share of negative sentiments, it does not have a significant correlation with the share of positive sentiments. However, it is negatively correlated with the real GDP growth rate, which has a more significant negative correlation with the share of negative sentiments and offensive and hateful speeches. For further analysis, the real GDP growth rate is also included.

### Explanatory models

A regression analysis at the country level is conducted to analyze the data more systematically. The dataset we use covers the years 2014–2019 and 11 countries specified in this study. First, a pooled regression is used to assess within as well as between-country variation. Then, the same model with countries in the subgroup is analyzed. Finally, the linear fixed effects model is used to eliminate time-invariant country differences.

As explanatory variables, we include three key factors (controlled variables) in the model: the real GPD growth rate, total unemployment rate and the migrant stock. We use the migrant stock per 100 thousand population in the respective country in the regression models to avoid biases due to the country size differences. The regression is thus specified as:

1 $$\begin{aligned} {\textit{Share Of posts}}_{ct}&= \alpha _c + \tau _t + \beta {\textit{RealGDPGrowthRate}}_{ct}\nonumber \\&\quad + \delta {\textit{UNEMP}}_{ct} + \upsilon {\textit{MIGR}}_{ct} + \epsilon _{ct} \end{aligned}$$

where *c* indicates the country and *t* is the year, $\alpha _{c}$ are country fixed effects and $\tau _{t}$ are year fixed effects. In the pooled regression, these two terms are replaced by a simple constant $\alpha$.

**Table 7:** Pooled linear regression model with explanatory model (***$p<0.01$, **$p<0.05$, *$p<0.1$)

	(1) Share of posts with negative sentiment	(2) Share of offensive posts	(3) Share of hateful posts	(4) Share of posts with negative sentiment	(5) Share of offensive posts	(6) Share of hateful posts
Yearly first-time asylum seekers per 100k population	− 1.355e−05 (1.67e−05)	− 8.085e−07 (1.66e−06)	− 1.133e−05* (6.51e−06)			
Total migrant stock per 100k population	− 2.032e−06** (9.18e−07)	− 8.085e−07*** (1.66e−06)	− 1.573e−06*** (3.12e−07)	− 2.759e−06* (1.43e−06)	− 2.027e−07* (1.1e−07)	− 1.425e−06*** (4.53e−07)
Total unemployment rate (15–74)	− 0.0025 (0.001)	− 0.0002 (0.000)	− 0.0010* (0.001)	− 0.0021 (0.002)	− 0.0002 (0.000)	− 0.0010 (0.001)
Real GDP growth rate	− 0.0018 (0.005)	7.467e−05 (0.001)	− 0.0019 (0.002)	− 0.0029 (0.005)	0.0001 (0.001)	− 0.0020 (0.002)
Yearly immigration flow per 100k population				1.228e−05 (2.13e−05)	− 1.521e−06 (1.76e−06)	− 3.946e−06 (6.96e−06)
Constant	0.1176*** (0.027)	0.0143*** (0.003)	0.0689*** (0.011)	0.1082*** (0.030)	0.0150*** (0.003)	0.0692*** (0.012)
Observations	66	66	66	66	66	66
*R*-squared	0.072	0.119	0.163	0.070	0.122	0.145

Table 7 provides results for the pooled regression. Each column is one regression model with a different outcome variable, as indicated at the top of the column. Column (1) shows that the negative correlation shown in Table 6 also persists when controlling for migrant stock, unemployment rate, and real GDP growth rate, as well as variable the number of asylum seekers. However, the factors are not statistically significant except for migrant stock. Column (1) thus shows that countries see a lower share of negative posts with larger migrant stock. The same goes for Column (2), i.e., there are fewer offensive posts within countries with more immigrants. Column (3) shows that aside from the statistical significance of the negative correlation between migrant stock and the share of hateful posts, the countries see a lower share of hateful posts when there are larger numbers of first-time asylum applicants. In Column (4), (5), (6), immigrant inflows are used instead of asylum requests; there is no significant pattern as in Column (3). It does not seem to be immigration flows per se but precisely the number of asylum seekers that drives the observed pattern. However, the significance of the migrant stock persists. Furthermore, overall, $R^{2}$ is relatively small throughout, indicating that the factors included in the model explain less than 20% of the variation in the data—and sometimes less than 10%. This is striking as $R^{2}$ shown here covers both static differences between countries (between dimensions) and the country-specific changes over time (within dimension).

**Table 8:** Pooled linear regression model with subgroup analysis (***$p<0.01$, **$p<0.05$, *$p<0.1$)

Outcome variable	(1) Share of posts with negative sentiment	(2) Share of offensive posts	(3) Share of hateful posts
Subsample	Below median total/non-EU immigrant stock	Below median total/non-EU immigrant stock	Below median total/non-EU immigrant stock
Yearly first-time asylum seekers per 100k population	3.826e−06 (1.57e−05)	5.023e−07 (1.96e−06)	− 6.028e−06 (7.45e−06)
Total migrant stock per 100k population	− 1.796e−06 (2.1e−06)	− 4.547e−07** (1.94e−07)	− 2.985e−06*** (5.93e−07)
Total unemployment rate (15–74)	0.0087 (0.007)	6.661e−05 (0.001)	0.0027 (0.004)
Real GDP growth rate	0.0046 (0.008)	− 0.0003 (0.001)	− 0.0032 (0.003)
Yearly immigration flow per 100k population	4.112e−07** (1.54e−07)	1.36e−08 (1.93e−08)	− 7.911e−08 (6.13e−08)
Constant	− 0.0415 (0.058)	0.0119* (0.006)	0.0641** (0.027)
Observations	30	30	30
*R*-squared	0.277	0.182	0.391

Next, whether the observed patterns are in line with the contact hypothesis is examined, that the local population processes immigrant inflows in a given year differently depending on how much experience they have with migrants. For analysis, the sample is split at the median of the migrant stock in Table 8. After splitting, the analyzed countries are Austria, Hungary, the Netherlands, Poland, and Sweden. In these countries, both total migrant stocks and non-EU migrant (which are more similar to asylum seekers since these come from non-EU countries) stocks are below the median. The table shows that there are fewer hateful speeches in countries with more migrants, receiving more migrants and asylum seekers. For posts with negative sentiments, the positive correlation with immigration flow is more statistically significant than its negative correlation with migrant stock. While, regarding the offensive posts, it is the opposite, i.e., overall, the observed pattern that countries with more migrants have lesser negative, offensive, and hateful posts do persist in all sub-samples. Moreover, $R^{2}$ and the statistical significance of the coefficient of interest are markedly higher than in Column (3) compared to Column (1) and Column (2), which suggests that the observed negative relationship between hateful speeches and migrant stock is more relevant compared to the negative and offensive posts.

**Table 9:** Linear fixed effects model accounting for time-invariant country differences (***$p<0.01$, **$p<0.05$, *$p<0.1$)

Outcome variable	(1) Share of posts with negative sentiment	(2) Share of offensive posts	(3) Share of hateful posts	(4) Share of posts with negative sentiment	(5) Share of offensive posts	(6) Share of hateful posts
Yearly first-time asylum seekers per 100k population	− 3.59e−05*** (8.037e−06)	− 1.914e−06* (1.108e−06)	− 2.023e−05** (7.947e−06)	2.256e−06 (8.169e−06)	1.638e−06 (1.276e−06)	2.256e−06 (8.169e−06)
Total migrant stock per 100k population	− 1.187e−06*** (3.182e−07)	− 2.299e−07*** (6.624e−08)	− 1.255e−06*** (1.535e−07)	− 4.747e−07 (3.403e−06)	2.392e−07 (5.7e−07)	− 4.747e−07 (3.403e−06)
Total unemployment rate (15–74)	− 0.0031*** (0.0007)	− 0.0002 (0.0002)	− 0.0014*** (0.0003)	− 0.0015	0.0009	− 0.0015
Real GDP growth rate	0.0056 (0.0051)	0.0005 (0.0005)	0.0006 (0.0024)	0.0055 (0.0071)	7.489e−05 (0.0014)	0.0055 (0.0071)
Country-specific linear time trends	No	No	No	Yes	Yes	Yes
Observations	66	66	66	66	66	66
*R*-squared (within)	− 0.1531	− 0.1165	− 0.1442	− 0.0424	0.0318	0.0380
Number of country fixed effects	11	11	11	11	11	11

The patterns observed in the pooled sample could come from time-invariant differences between countries, such as culture, the general willingness of populations to welcome migrants, or differences in social media use. Such unobserved factors could be correlated with the driving intention of asylum seekers to go to a specific EU member state, resulting in an omitted variable bias. To rule out that persistent differences between countries are the drivers of the negative correlation between asylum applications and the share of posts with negative sentiments, and offensive or hateful speeches, we eliminate time-invariant country differences in Table 9.

The results in Column (1), (2), and (3) show that the negative correlation in the previous specification (especially in Table 7) persists after controlling for time-invariant country differences. However, the within-$R^2$ in these columns are negative, which means the model does not fit the data well. To analyze at what level the relevant variation determining differences in attitudes as measured by our outcome variables occurs, we add country-specific linear time trends in Column (4), (5), and (6). These lead to better within-$R^2$s. Comparing columns (1) and (4), which rely on the same outcome variable, and columns (2) and (5), columns (3) and (6), respectively, we can conclude that differences over time in 11 EU countries explain relatively little. Adding the linear country-specific time trends improves the model, suggesting that country-specific factors outside the explanatory factors explain changes to some extent. However, the overall share of explained variation is still only about 3%. Hence, much of attitude changes over time are hard to pin down.

The pattern between asylum requests and prior contact with the migrants and negative sentiments that we have observed in the pooled sample thus seems not to be causal. Instead, differences in the attitudes of Twitter posts can be seen across the 11 EU countries over time. Factors such as unemployment rates and real GDP growth rate explain relatively little. In particular, these factors do not explain the share of offensive posts well.

## Use cases and sustainability

MGKB can facilitate various studies in interdisciplinary research, especially in sociology, social sciences, and economics. First, the various application scenarios facilitated by MGKB are illustrated and explained in this section. Secondly, with the ongoing evolving tweets regarding migrations, the continuous maintenance and extension of MGKB are necessary, which will be explained in detail in the second part of this section.

### Scenarios and queries

MGKB can facilitate several application scenarios, some of which are detailed in the following:

*Usefulness in social sciences* MGKB links potential social and economic driving factors affecting the public attitudes toward migration with the results from semantic analysis such as topic modeling, sentiment analysis, and hate speech detection. Moreover, it will continuously incorporate more factors according to recently established research in social sciences (Drazanova 2020) in this matter. Based on MGKB, a more detailed analysis of the potential factors driving the public attitudes toward migrations is conducted in this study (cf. Sect. 5, such as applying statistical methods to determine which factors are essential to determine the negative sentiments or offensive and hateful speeches, which can further facilitate research in social sciences.

For example, to understand potential factors driving the public attitudes toward migrations in the UK, MGKB can be queried for extracting the potential driving factors and the number of tweets in different sentiments and offensive/hateful speech from the UK over the years in Table 10(a). The query result in Table 10(b) shows (1) the negative correlation between disposal income and total unemployment rate, which is reflected by the pairwise correlation analysis in Table 11; (2) the positive correlation between negative sentiments and offensive/hateful speeches, which is statistically significant in Table 11; (3) there are more negative sentiments and offensive/hateful speeches when there are more people unemployed and less disposable income per inhabitant, however, such positive correlation is only statistically significant regarding hateful speeches, as shown in Table 11. Moreover, the pairwise correlation analysis shows that, in the UK, as the total unemployment rate is low, there are more migrants in the country, and there are more asylum requests. In summary, more hateful speeches occur when there are fewer migrant stock and fewer asylum requests, which is in line with the contact hypothesis that people tend to be more positive toward migrants when they are already in contact with migrants.

**Table 10:** The query and result for extracting the potential driving factors and the number of tweets in different sentiments and offensive/hateful speeches from the UK over the years 2013–2020

(a) SPARQL query Q1								
SELECT ?Year ?RealGDPGrowthRate ?TotalUnemploymentRate ?DisposableIncome								
(SUM(IF (?atti=mgkb:offensive, 1, 0)) AS ?Offensive)								
(SUM(IF (?atti=wna:hate, 1, 0)) AS ?Hateful)								
(SUM(IF (?atti=wna:negative-emotion, 1, 0)) AS ?Negative)								
(SUM(IF (?atti=wna:positive-emotion, 1, 0)) AS ?Positive)								
(COUNT(?tweet) AS ?TotalTweets)								
WHERE {								
?tweet fibo_fnd_rel_rel:isCharacterizedBy ?gdpr.								
?gdpr a fibo_ind_ei_ei:GrossDomesticProduct;								
schema:addressCountry “GB”; dc:date ?Year;								
fibo_ind_ei_ei:hasIndicatorValue ?RealGDPGrowthRate.								
?tweet fibo_fnd_rel_rel:isCharacterizedBy ?unemploy.								
?unemploy a mgkb:TotalUnemploymentRate;								
schema:addressCountry “GB”; dc:date ?Year;								
fibo_ind_ei_ei:hasIndicatorValue ?TotalUnemploymentRate.								
?tweet fibo_fnd_rel_rel:isCharacterizedBy ?income.								
?income a mgkb:DisposableIncome;								
fibo_ind_acc_cat:hasMonetaryAmount ?monetaryamount;								
dc:date ?Year; schema:addressCountry “GB”.								
?monetaryamount fibo_ind_acc_cat:hasAmount ?DisposableIncome.								
?tweet onyx:hasEmotionSet ?y.								
?y a onyx:EmotionSet; onyx:hasEmotion ?z.								
?z a onyx:Emotion; onyx:hasEmotionCategory ?atti.								
}GROUP BY ?Year ?RealGDPGrowthRate ?TotalUnemploymentRate ?DisposableIncome								
ORDER BY DESC(?Year)								

(b) The result								
Year	Real GDP growth rate	Total unemployment rate	Disposable income	Offensive	Hateful	Negative	Positive	Total tweets
2020	− 1.0	4.9	24,623	1298	5928	14,445	7095	89,768
2019	1.4	3.8	28,125	916	4698	11,165	7674	76,700
2018	1.3	4.0	26,989	31	126	161	841	5268
2017	1.7	4.3	26,122	38	115	232	259	2914
2016	1.7	4.8	24,961	62	299	537	386	5068
2015	2.4	5.3	24,219	185	951	2104	1158	16,160
2014	2.9	6.1	23,317	311	1865	3619	2033	25,012
2013	2.2	7.5	22,272	161	934	1453	1035	11,026

**Table 11:** Correlation matrix of factors used in multivariate analysis of potential determinants in the UK regarding public attitudes toward migrations based on (b) (***$p<0.01$, **$p<0.05$, *$p<0.1$)

	Share negative	Share positive	Share offensive	Share hateful	Total unemployment rate	Disposable income	Real GDP growth rate	Immigration flow per 100k	Asylum requests per 100k	Migrant stock per 100k
Share positive	− 0.7*	1								
Share offensive	0.86***	− 0.81**	1							
Share hateful	0.92***	− 0.56	0.78**	1						
Total unemployment rate	0.3	− 0.06	0.31	0.63*	1					
Disposable income	− 0.28	0.26	− 0.34	− 0.58	− 0.94 ***	1				
Real GDP growth rate	− 0.26	0.21	− 0.32	0.0	0.39	− 0.29	1			
Immigration flow per 100k	0.29	− 0.45	0.15	− 0.04	− 0.61	0.55	− 0.0	1		
Asylum requests per 100k	0.1	− 0.27	− 0.03	− 0.18	− 0.74*	0.72*	− 0.51	0.54	1	
Migrant stock per 100k	− 0.54	0.69	− 0.46	− 0.72	− 0.98 ***	0.92 ***	− 0.95 ***	− 0.1	0.36	1

*Analyzing risk of tension* There have been many studies (Ekman 2019; Conzo et al. 2021), showing that the anti-immigration discourses and portrayals in social media and news outlets have been more polarized and intensified over the last decades, which negatively influences the public perception of the immigrants and further raises the risk of tension between immigrants and the local residents which can also lead to violence. With an analysis of the dominating factors driving the public attitudes toward immigrants in the host countries, the policymakers can be advised accordingly to take measures for reducing the risk of tensions between the immigrants and the local residents. As shown in Table 11, the potential factors driving the public attitudes are the unemployment rate and migrant stocks. The programs to make both migrants and local residents employable within the country are essential to raising the employment rate. Moreover, assuring mutual contact between the migrants and local residents is also helpful in reducing the tension between the two parties.

*Relation between the global events and public attitudes toward migration* With the classified topics and linked entities in MGKB and the geographical and temporal information, the correlations between the events happening within a particular country and the changes in the attitudes over time can be built. For example, Table 12 shows query (a) and result (b) for extracting the number of hateful, negative, positive and total number of tweets regarding the topics containing “border” and “refugee” in the UK from 2013 to 2021. This specific topic includes the top topic words, i.e., work, refugee, covid, border, woman, long, uk, young, hard, poland, power, photooftheday, politic, foreign, deny, traveler, etc. The number of tweets aligns with the historical events. In 2019, the COVID-19 pandemic started, and more tweets discuss “refugee,” “COVID” and “border.” Specifically, the number of hateful and negative tweets and the total number of tweets peaked in 2020.

**Table 12:** The query and result for extracting the number of hateful, negative, positive tweets and the total number of tweets regarding the topics which contain keywords “border” and “covid” in the UK by temporal distribution

(a) SPARQL query Q2				
SELECT ?Year				
(SUM(IF (?atti=wna:hate, 1, 0)) AS ?Hateful)				
(SUM(IF (?atti=wna:negative-emotion, 1, 0)) AS ?Negative)				
(SUM(IF (?atti=wna:positive-emotion, 1, 0)) AS ?Positive)				
(COUNT(?tweet) AS ?Total) WHERE {				
?tweet dc:subject ?topic;				
onyx:hasEmotionSet ?y ;				
schema:location ?location;				
dcterms:created ?createdDate.				
?location schema:addressCountry “GB”.				
BIND( SUBSTR(?createdDate,1,4 ) AS ?Year).				
?y a onyx:EmotionSet;				
onyx:hasEmotion ?z.				
?z a onyx:Emotion;				
onyx:hasEmotionCategory ?atti.				
?topic a sioc_t:Category ;				
rdfs:label ?topicLabel.				
FILTER( REGEX(?topicLabel, “border”, “i”)				
|| LCASE(STR(?topicLabel))=“refugee”).				
}GROUP BY ?Year ORDER BY DESC(?Year)				

(b) The result				
Year	Hateful	Negative	Positive	Total
2021	58	140	129	1186
2020	108	321	294	2714
2019	79	191	242	1742
2018	2	2	19	102
2017	5	15	10	132
2016	9	23	9	184
2015	26	45	43	390
2014	36	102	59	754
2013	17	36	29	342

*Entity-centric analysis* With the help of mapped entities in Wikidata, sentiment analysis, and hate speech detection, MGKB can facilitate various entity-centric analyses. Table 13 shows the query (a) and the first 15 results (b) for extracting the top occurring entities concerning refugees linked to Wikidata in the tweets and the tweets’ sentiments and offensive/hate speeches. For example, for tweets concerning “Zaatari refugee camp,” most tweets contain normal languages and neutral sentiment, i.e., 71 and 49 tweets, while 18 of them have negative sentiments. As shown in the table, the most frequently occurring entity containing “refugee” is the entity “United Nations High Commissioner for Refugees” (UNHCR), which indicates the high involvement of promotion tweets from UNHCR. Table 14 shows the query (a) and result (b) for retrieving a list of top 10 entity labels containing “refugee” and their number of detected entity mentions. While Table 15 shows the query (a) and result (b) for identifying the sentiments and hate speech languages of the public over time by searching entities defining “Refugees.” As shown in Table 15(b), overall, there are more negative tweets and hate speeches concerning “refugees” from 2019 to 2021 compared to the previous years. The overall distribution is reflected by the temporal distribution of sentiments and hate speeches in Figs. 5 and 6.

**Table 13:** The query and the first 15 results for extracting top occurring entities concerning “refugees” linked to Wikidata in the tweets and their sentiments and offensive/hate speeches

(a) SPARQL query Q3			
SELECT ?EmotionCategory ?EntityMention ?Entities (COUNT(?tweet) AS ?NumOfTweets) WHERE{			
?tweet schema:mentions ?entity.			
?entity a nee:Entity; nee:hasMatchedURI ?Entities.			
?Entities a rdfs:Resource; rdfs:label ?EntityMention.			
FILTER( REGEX(?EntityMention, “refuge”, “i”) || LCASE(STR(?EntityMention))=“refuge”).			
?tweet onyx:hasEmotionSet ?y.			
?y a onyx:EmotionSet; onyx:hasEmotion ?z.			
?z a onyx:Emotion; onyx:hasEmotionCategory ?EmotionCategory.			
} GROUP BY ?EmotionCategory ?EntityMention ?Entities ORDER BY DESC(?NumOfTweets) LIMIT 15			

(b) The result			
Emotion category	Entity mention	Entities (Wikidata)	NumOfTweets
normal speech	United Nations High Commissioner for Refugees	http://www.wikidata.org/entity/Q132551	5401
neutral sentiment	United Nations High Commissioner for Refugees	http://www.wikidata.org/entity/Q132551	3680
negative sentiment	United Nations High Commissioner for Refugees	http://www.wikidata.org/entity/Q132551	2134
hate speech	United Nations High Commissioner for Refugees	http://www.wikidata.org/entity/Q132551	981
positive sentiment	United Nations High Commissioner for Refugees	http://www.wikidata.org/entity/Q132551	761
offensive speech	United Nations High Commissioner for Refugees	http://www.wikidata.org/entity/Q132551	193
normal speech	Zaatari refugee camp	http://www.wikidata.org/entity/Q8063414	71
normal speech	Refugee	http://www.wikidata.org/entity/Q131572	69
neutral sentiment	Zaatari refugee camp	http://www.wikidata.org/entity/Q8063414	49
neutral sentiment	Refugee	http://www.wikidata.org/entity/Q131572	43
normal speech	Refugee Week	http://www.wikidata.org/entity/Q7307664	41
normal speech	Refugee shelter	http://www.wikidata.org/entity/Q2681882	33
normal speech	North of England Refugee Service	http://www.wikidata.org/entity/Q16211246	26
neutral sentiment	Refugee Week	http://www.wikidata.org/entity/Q7307664	25
neutral sentiment	North of England Refugee Service	http://www.wikidata.org/entity/Q16211246	23

**Table 14:** The query and result for retrieving a list of top 10 entity labels containing “refugee” and its frequency of detected entity mentions

(a) SPARQL query Q4	
SELECT ?EntityLabel (COUNT(?EntityLabel) AS	
?NumOfEntityMentions) WHERE{	
?tweet schema:mentions ?entity.	
?entity a nee:Entity;	
nee:hasMatchedURI ?uri.	
?uri a rdfs:Resource;	
rdfs:label ?EntityLabel.	
FILTER( REGEX(?EntityLabel, “refugee”, “i”) ||	
LCASE(STR(?EntityLabel))=“refugee”).	
}GROUP BY ?EntityLabel	
ORDER BY DESC(?NumOfEntityMentions) LIMIT 10	

(b) The result	
EntityLabel	NumOf Entity Mentions
United Nations High Commissioner for Refugees	6575
Zaatari refugee camp	72
Refugee	69
Refugee Week	43
Refugee shelter	34
North of England Refugee Service	29
Kiryandongo Refugee Settlement	13
Refugee Blues	11
Nakivale Refugee Settlement	10
Refugee Action	8

**Table 15:** The query and result for identifying the negative and positive sentiments and hate speeches of the Public and the total number of tweets over time by searching entities defining “Refugees”

(a) SPARQL query Q5				
SELECT ?Year (SUM(IF (?atti=wna:hate, 1, 0)) AS ?Hateful)				
(SUM(IF (?atti=wna:negative-emotion, 1, 0)) AS ?Negative)				
(SUM(IF (?atti=wna:positive-emotion, 1, 0)) AS ?Positive)				
(count(?tweet) as ?TotalTweets) WHERE {				
?tweet schema:mentions ?entity;				
dcterms:created ?createdDate.				
?entity a nee:Entity;				
nee:hasMatchedURI ?uri.				
?uri a rdfs:Resource;				
rdfs:label ?x.				
BIND( SUBSTR(?createdDate,1,4 ) AS ?Year).				
FILTER( REGEX(?x, “refugee”, “i”)				
|| LCASE(STR(?x))=“refugee”).				
?tweet onyx:hasEmotionSet ?y.				
?y a onyx:EmotionSet;				
onyx:hasEmotion ?z.				
?z a onyx:Emotion;				
onyx:hasEmotionCategory ?atti.				
}GROUP BY ?Year ORDER BY DESC(?Year)				

(b) The result				
Year	Hateful	Negative	Positive	Total
2021	133	328	120	1888
2020	341	742	282	4522
2019	398	905	336	5534
2018	7	14	12	170
2017	4	15	3	112
2016	7	16	6	124
2015	19	52	23	404
2014	60	97	53	826
2013	35	55	25	494

### Sustainability, maintenance, and extensibility

Since the issue of migrations is highly controversial and salient in Europe, the reuse of MGKB is anticipated. It is, therefore, crucial to ensure the sustainability of the KB. While the project mentioned above and the IT tool are still under development, MGKB is expected to be advertised through interdisciplinary networks and events. Besides, MGKB will be updated and maintained on Zenodo and GitHub pages using permanent URIs, making it citable and findable.

MGKB will be maintained through the continuous process of crawled migration-related tweets through the Twitter API. The topic modeling will be periodically repeated on the expanded Twitter data to capture new topics. The semantic annotations resulting from sentiment analysis and hate speech detection will be updated with the state-of-the-art language models and neural networks and evaluated on the benchmark datasets in the corresponding tasks. To that end, MGKB will be incrementally expanded with newer tweets, and the semantic annotations are ensured of correctness. Moreover, to ensure that the data reflects user intent and the current state of content on Twitter, we will follow batch compliance^16^ periodically to delete data that is restricted from the users.

The MGKB ontology is also extensible; it can further enrich with additional information about the tweets, such as conversational relations between Twitter users, and incorporates social and economic indicators on a finer-grained level. Moreover, augmenting the current version with multilingual analysis is essential to capture a broader range of European countries, and, accordingly, the current schema is to be extended with multilingual ontologies. With the growing attention of MGKB in interdisciplinary research fields, such extensions are necessary to facilitate further research regarding migrations. Furthermore, to enable real-time observation and prediction of migration flows and patterns, Twitter stream analysis will be integrated into the current pipeline.

## Discussion, conclusion and future work

In this study, a KB of migration-related tweets is presented. The tweets are filtered using neural-based topic modeling, sentiment analysis is performed based on BERT, and attention-based hate speech detection is performed. Moreover, the BERT-based entity linking to Wikipedia and Wikidata is performed. MGKB extends the ontology defined by TweetsKB by adding the geographical information of tweets, the social and economic indicators of the European countries, and the results from the analyses. MGKB can provide a better understanding of public attitudes toward migrations. The detailed analysis of the public attitudes and the social and economic indicators incorporated in the MGKB shows that the countries with more migrants have fewer hateful and negative tweets. The other potential driving factors affecting public attitudes, such as unemployment rate, disposable income, and real GDP growth rate, are analyzed as well. Afterward, use-cases and SPARQL queries are defined and explained. Finally, MGKB as a resource is published using FAIR principles and will be continuously updated and maintained.

In the current version of MGKB, the focus is solely on the tweets in English; the distribution of the corpus is therefore highly skewed by the tweets from the UK. While focusing on the destination countries in Europe, there is already a wide variety of languages that need attention. The multilingual analysis will be integrated into the pipeline for future work, including selected official languages used across European countries. Moreover, regional social and economic indicators will be incorporated into MGKB instead of current country-level indicators. Accordingly, the MGKB will be extended with multilingual and more extensive social and economic indicator ontologies. Additionally, stance detection would also be performed based on a multilingual setting. Moreover, the current version of the pipeline for MGKB provides a basis for the automated updates of the MGKB with the help of Twitter stream analysis approaches in various languages.

## Footnotes

1 https://w3id.org/mgkb/codes.

2 https://w3id.org/mgkb.

3 https://w3id.org/mgkb/sparql.

4 https://zenodo.org/record/5206826.

5 https://www.oecd.org/about/members-and-partners/.

6 http://sentistrength.wlv.ac.uk/.

7 Keywords: https://bit.ly/3APiKIw, Hashtags: https://bit.ly/2W1TMXk.

8 https://w3id.org/mgkb#query_1.

9 https://w3id.org/mgkb#topics_etm.

10 https://w3id.org/mgkb#stats_plots.

11 Twitter US Airline Sentiment: https://bit.ly/3953GxQ.

12 The 2019/08/01 Wikipedia dump, which is downloadable in its raw format from http://dl.fbaipublicfiles.com/BLINK/enwiki-pages-articles.xml.bz2.

13 https://bit.ly/37ExO2y.

14 https://migrationskb.github.io/MGKB/migrationsKB/documentation.html.

15 https://bit.ly/3N8vzUj.

16 https://developer.twitter.com/en/docs/twitter-api/compliance/batch-compliance/introduction.

## Declarations

### Data availability

The datasets generated during and analyzed during the current study are available in the GitHub repository, https://w3id.org/mgkb/codes, and data dumps are made available on Zenodo, https://zenodo.org/record/5206826. The MGKB can be directly accessed via a SPARQL endpoint, https://w3id.org/mgkb/sparql. The extended ontology is hosted on the website http://w3id.org/mgkb, which also contains documentation, more figures, query examples, and statistics.
